# Kirigami-based stretchable lithium-ion batteries

**DOI:** 10.1038/srep10988

**Published:** 2015-06-11

**Authors:** Zeming Song, Xu Wang, Cheng Lv, Yonghao An, Mengbing Liang, Teng Ma, David He, Ying-Jie Zheng, Shi-Qing Huang, Hongyu Yu, Hanqing Jiang

**Affiliations:** 1 School for Engineering of Matter, Transport and Energy, Arizona State University, Tempe, AZ 85287, USA; 2 Desert Vista High School, Phoenix, AZ 85048, USA; 3 MOE Key Lab of Disaster Forecast and Control in Engineering, Jinan University, Guangzhou 510632, China; 4 School of Electrical, Computer and Energy Engineering, Arizona State University, Tempe, AZ 85287, USA; 5 School of Earth and Space Exploration, Arizona State University, Tempe, AZ 85287, USA

## Abstract

We have produced stretchable lithium-ion batteries (LIBs) using the concept of kirigami, i.e., a combination of folding and cutting. The designated kirigami patterns have been discovered and implemented to achieve great stretchability (over 150%) to LIBs that are produced by standardized battery manufacturing. It is shown that fracture due to cutting and folding is suppressed by plastic rolling, which provides kirigami LIBs excellent electrochemical and mechanical characteristics. The kirigami LIBs have demonstrated the capability to be integrated and power a smart watch, which may disruptively impact the field of wearable electronics by offering extra physical and functionality design spaces.

Energy storage devices, such as supercapacitors and lithium-ion batteries (LIBs) that are able to sustain large strains (much greater than 1%) under complex deformations (for instance, bending, tension/compression, and torsion) are indispensable components for flexible, stretchable electronics, and recently emerging wearable electronics, such as flexible displays^1^,^2^,^3^,^4^, stretchable circuits^5^, hemispherical electronic eyes^6^, and epidermal electronics^7^. Various approaches have been employed to achieve flexible and stretchable energy storage devices, such as thin film based bendable supercapacitors^8^,^9^,^10^,^11^ and batteries^10^,^12^,^13^,^14^,^15^,^16^, buckling-based stretchable supercapacitors^17^,^18^, and island-serpentine-based stretchable LIBs^19^. Recently, an origami-based approach was adopted to develop highly foldable LIBs, where standard LIBs were produced followed by designated origami folding^20^. The folding endows the origami LIB with a high level of foldability by changing the LIB from a planar state to a folded state. However, the previously developed origami-based foldable devices^20^,^21^ have two disadvantages. First, their foldability is limited from the folded state to the planar state. Although it can be tuned by different folding patterns, the same constraint is still prescribed by the planar state. Second, the folded state involves uneven surfaces, which introduces inconvenience when integrating with planar systems, though this issue can be somewhat circumvented. The approach introduced here combines folding and cutting, by the name of kirigami, to define patterns that form an even surface after stretching and the stretchability is not limited by the planar state. The folding and cutting lead to high level of stretchability through a new mechanism, “plastic rolling”, which has not yet been discovered and utilized in the stretchable electronics/systems. The LIBs were produced by the standard slurry coating (using graphite as an anode and LiCoO_2_ as a cathode) and packaging procedure, followed by a designated folding and cutting procedure to achieve a particular kirigami pattern. Kirigami batteries are also compatible with emerging battery fabrication skills such as direct printing or painting^22^. Following kirigami patterns, the printed or painted kirigami batteries is expected to perform similarly as batteries fabricated in conventional way. Over 150% stretchability has been achieved and the produced kirigami LIBs have demonstrated the ability to power a Samsung Gear 2 smart watch, which shows the potential applications of this approach. The kirigami-based methodology can be readily expanded to other applications to develop highly stretchable devices and thus deeply and broadly impact the field of stretchable and wearable electronics.

## Results

### Battery design using Kirigami patterns

**Figure 1:**
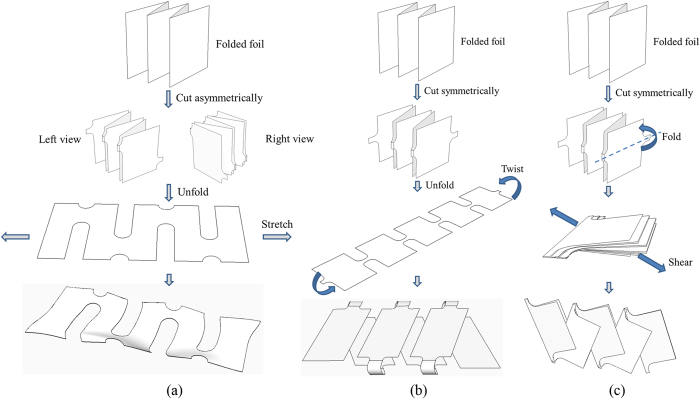
Illustrations of three kirigami patterns. (**a**) A zigzag-cut pattern, where the out-of-plane deformation occurs to accommodate stretching. (**b**) A cut-N-twist pattern, where the rotation is involved to accommodate stretching and no out-of-plane deformation. (**c**) A cut-N-shear pattern, where the packing density doubles compared with that of the cut-N-twist pattern. Rotation is involved to accommodate stretching and no out-of-plane deformation.

Three kirigami patterns are utilized, as illustrated in Fig. 1, with (a) a zigzag-cut pattern, (b) a cut-N-twist pattern, and (c) a cut-N-shear pattern. The zigzag-cut pattern (Fig. 1a) represents one of the most commonly seen kirigami patterns and is produced by cutting a folded stack of foil asymmetrically between the neighboring creases, which creates zigzag-liked cuttings in the longitudinal direction. The zigzag pattern can be stretched beyond its length in the planar state, which is the advantage of kirigami. To accommodate stretching, the out-of-plane deformation (or equivalently, buckling) occurs at the vicinity of cuts. The level of stretchability depends on the length of the cut and is a function of buckling amplitude. To eliminate the out-of-plane deformation, one of the advantages of kirigami compared with the origami pattern, the cut-N-twist pattern (Fig. 1b) is utilized, in which a folded stack of foil is symmetrically cut at all creases, and then unfolded to a planar state, followed by twisting at the two ends. The twisted structure is shown in the bottom panel of Fig. 1b and analogous to a twisted telephone cord. This pattern represents a locked structure in the sense that the out-of-plane deformation, induced by stretching, is constrained and rotation occurs at the cuts to accommodate stretching. The packing density of cut-N-twist pattern is defined by the width of each face. To increase the packing density, the cut-N-shear pattern (Fig. 1c) is introduced, where folding is employed after symmetric cutting and then the folded structure is subjected to shear, thus the packing density doubles compared with that for the cut-N-twist pattern. The stretching is also achieved by the rotations of the cuts and no out-of-plane deformation is involved.

Now we demonstrate kirigami LIBs, specifically the LIBs using cut-N-twist here and cut-N-shear and zigzag-cut patterns in the Supporting Information. Conventional materials and approaches of LIBs preparation were used, with graphite (Fisher Scientific Inc.) and LiCoO_2_ (LCO, MTI Corp.) as the anode and cathode active materials, respectively. Conventional slurries of these active materials were prepared and used to coat the current collectors, where copper (Cu) and aluminum (Al) served as the anode and cathode current collectors, respectively. The Cu and Al current collectors were first cut to the three patterns (Fig. 1) based on the specific geometries as provided in the Supplementary Fig. S1, followed by slurry coating. To prepare packaging, polypropylene (Celgard 2500) as separator and aluminized polyethylene (PE) (Sigma-Aldrich) as packaging materials were also cut using the same kirigami pattern; thus all the layers of a LIB have the same pattern. Then these layers were perfectly laminated in the order of packaging material, cathode electrode, separator, anode electrode, and packaging material (Supplementary Fig. S2), and delivered to an Argon-filled glovebox for packaging. The impulse sealer was used to seal the sides of the each cell (Supplementary Fig. S1) except one side for the electrolyte (1 M LiPF_6_ in EC:DMC:DEC (1:1:1), MTI Corp.) injection, followed by sealing the last side of the battery cell. The key to achieve excellent packaging is that the cutting of all layers of a battery cell must be uniform and the alignment must be perfect. We have designed a customized puncher for cutting. The cutting quality can be significantly improved by using laser cutting in the future.

### Electrochemical and mechanical characteristics

**Figure 2:**
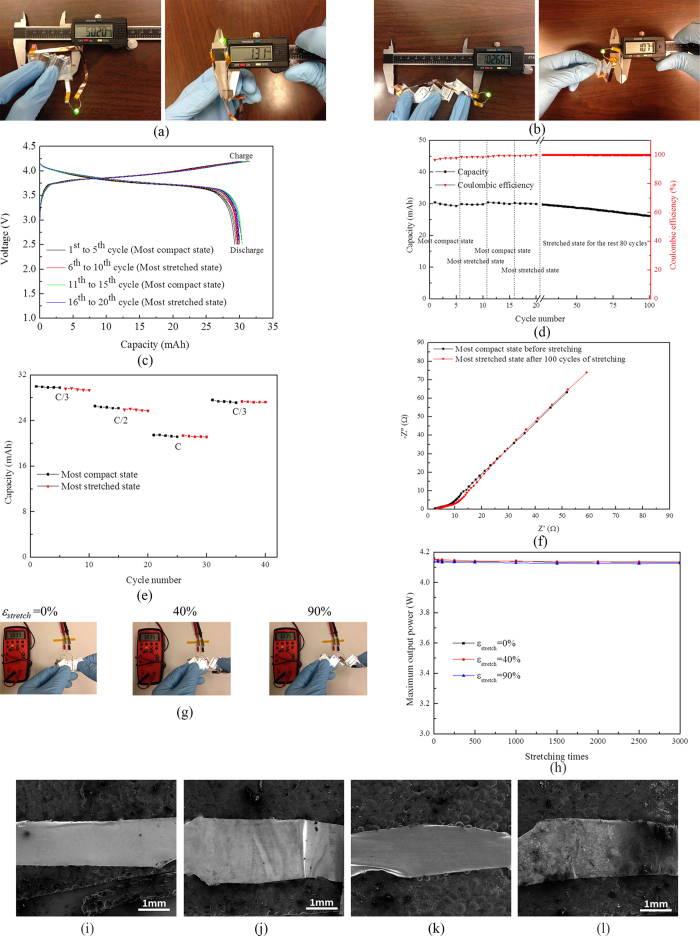
Electrochemical and mechanical characterizations of a kirigami lithium-ion battery (LIB) using cut-N-twist pattern. (**a**) Photograph of a LIB at its most compact state. (**b**) Photograph of a LIB at its most stretched state. (**c**) Galvanostatic charge and discharge at the most compact state (1^st^ to 5^th^ cycles), the most stretched state (6^th^ to 10^th^ cycles), the most compact state again (11^th^ to 15^th^ cycles), and the most stretched state again (16^th^ to 20^th^ cycles) under C/3 charge/discharge rate. The mass loading of LiCoO_2_ (LCO) (specific capacity 145 mAh g^−1^) and graphite (specific capacity 372 mAh g^−1^) were 95 mg and 255 mg, respectively, which gave LIB the capacity of 30 mAh. (**d**) Energy capacity (left axis, black) and Coulombic efficiency (right axis, red) as a function of cycle number for C/3 charge/discharge rate. The mass accounts for all the materials involved in a cell, which is 1.49 g. (**e**) Rate performance when the charge/discharge rates varied from C/3, C/2, to C, and C/3 again for both compact and stretched states. (**f**) Electrochemical impedance spectroscopy (EIS) analysis during the first discharge cycle at the most compact state before stretching and stretched state after 100 stretching cycles. EIS studies were performed by applying a small perturbation voltage of 5 mV in the frequency range of 0.1 Hz to 100 kHz. Typical impedance spectrum, with high-to-middle frequency range flat curve and a relative straight line representing the low frequency range, was observed. No obvious semicircle was observed because of the low internal resistant. There are not significant changes in the impedance before and after mechanical deformation. (**g**) Photograph of stretching a kirigami LIB while it was connected to a voltmeter. (**h**) Maximum output power of the kirigami LIB as a function of stretchability over 3,000 cycles of stretches. (**i**) Scanning electron micrographs (SEM) of anode current collector Cu at the cut before charge. (**j**) SEM of anode current collector Cu at the cut after discharge and 100 cycles of stretching. (**k**) SEM of cathode current collector Al at the cut before charge. (**l**) SEM of cathode current collector Al at the cut after discharge and 100 cycles of stretching.

Figure 2 shows electrochemical and mechanical characterization results for LIBs using cut-N-twist pattern. Using the most compact state (Fig. 2a) as the reference, Fig. 2b, displaying the LIB at its most stretched state, shows that the stretchability of a kirigami LIB is over 100%. It should be noted that there is no significant change on the thickness of this LIB between the most compact state (*h* = 1.31 mm) and at the most stretched state (*h* = 1.07 mm). Figure 2c shows the electrochemical cycling results of a kirigami LIB at its most compact state (for the 1^st^ to 5^th^ cycles), followed by that at its most stretched state (for the 6^th^ to 10^th^ cycles), then that at its most compact state again (for the 11^th^ to 15^th^ cycles), and then that at its most stretched state again (for the 16^th^ to 20^th^ cycles), and finally that at its most stretched state (for the 21^st^ to 100^th^ cycles) under C/3 charge/discharge rate. Well-defined plateaus at around 3.7 V are observed along with fairly stable charge/discharge behaviors under compact and stretched states. The present mass loading (see caption of Fig. 2c) gives this kirigami LIB 30 mAh energy capacity. Figure 2d shows reasonable cyclic stability of the LIBs up to 100 cycles with over 85% capacity retention and 99.8% Coulombic efficiency. It should be emphasized that this result represents the stability of this kirigami LIB at mixed states, i.e., both compact and stretched states. Figure 2e shows the rate performance of this kirigami battery when the charge/discharge rate varied in the sequence of C/3, C/2, C and C/3 again at both compact and stretched state. When discharge rates increase, as expected, the capacity decreases from 29.3 mAh for C/3 rate to 26.5 mAh for C/2 rate, and 21.4 mAh for discharge rate C. However, the capacity recovered to the 27.6 mAh when the discharge rate resumed to C/3 after 30 cycles charge/discharge at the both compact the stretched state under varies C-rates, which indicates great rate performance of this kirigami battery. Figure 2f provides the results for electrochemical impedance spectroscopy (EIS) studies during the first discharge cycle at the most compact state before stretching and stretched state after 100 cycles of mechanical stretching. No significant changes in the impedance were found.

The mechanical characteristics of the fully charged kirigami LIB using cut-N-twist are then examined. As shown in Fig. 2g, at different stretchability, the output voltage remained steady at 3.83 V. Supplementary Movie S1 shows the dynamic process of this deformation. Figure 2h shows the maximum output power of the kirigami LIB as a function of stretchability, *ε*_*stretch*_, under different cycles of stretching. Here the internal resistance of the battery was measured to be about 1.8 Ω Over 3,000 stretching cycles and a stretchability *ε*_*stretch*_ of up to 90%, it is found that the output power is stable and shows no noticeable decay. The maximum output power is 4.1 W and is sufficient to operate commercial light-emitting diodes (LEDs). As shown in the Supporting Information (Supplementary Figs. S3 and Supplementary Movie S1), LEDs driven by this kirigami LIB do not show noticeable dimming upon cyclic stretching for a few hours. Ultimate tensile strength of LIBs using cut-N-twist pattern is 13.3 MPa with load frame Instron-Model 4411.

Figure 2i,j show the scanning electron micrographs (SEMs) for the anode current collectors (e.g., Cu foil) at the cuts before charging, and after discharge and 100 cycles of mechanical deformation. The similar SEM images are given for the cathode current collectors (e.g., Al foils) in Fig. 2k,l. There are no cracks after cyclic mechanical stretching, which contribute to the robust electrochemical and mechanical characterizations.

### Numerical calculation of crack and plastic behavior

**Figure 3:**
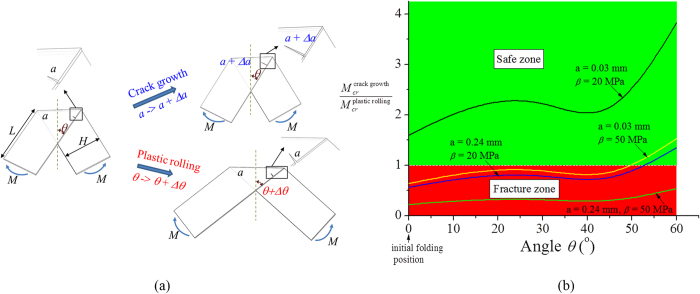
Theoretical analysis of kirigami crack growth versus plastic rolling. (**a**) Illustration of the two deformation modes, i.e., crack growth and plastic rolling. (**b**) “Safe zone” and “fracture zone” that are characterized by the ratio of critical moments, i.e.,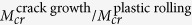, as a function of angle *θ*, for various α and *β*.

This phenomenon is consistent with the theoretical analysis and a simplified model is shown in Fig. 3a, where two pre-existing cracks are presumably caused by initial folding and/or cutting and located at the present positions when a pair of concentrated moment *M* is applied at the end of the strip with length *L* and width *H*. The concentrated moment *M* is used to characterize the applied stretching deformation that causes bending about the folding creases. Angle *θ* is used to denote the relative positions of two strips with *θ* = 0 for the initial folded position. When the moment *M* is applied, there exist two modes of deformation. The first mode causes the growth of the pre-existing cracks from *a* to *a* + *Δa*, while maintaining the angle *θ* unchanged, which refers as “crack growth”. The second mode leads to plastic deformation of the thin foil at the vicinity of the fold by altering *θ* to *θ* + *Δθ*, which is referred to as “plastic rolling”.

The critical condition for “crack growth” is given by the Griffith’s criterion. The driving force, or the release of the elastic energy due to the propagation of cracks, *Aσ*^2^*a*/*E*, equates the resistance of the crack growth, defined by 2*γ* Here *A* is a non-dimensional geometrical factor that depends on angle *θ*, i.e., *A* = *A*(*θ*); *σ* is the normal traction applied on the crack surface and related to the moment *M* by *σ* = 6*M*/*H*^2^; *E* is the elastic modulus; and *γ* [unit: Newton/meter] is the surface energy. Thus the critical moment for “crack growth” is given by 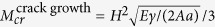. For “plastic rolling”, the rate of energy dissipation due to the plastic deformation during the rolling about the creases provides the resistance, given by 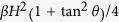; and the driving force is the rate of release of potential energy due to the increase of *θ*, given by *M*/2. Here *β* [unit: Newton/meter^2^] is the dissipated energy per unit area due to plastic rolling, which is related to the extent of the plastic deformation (i.e., hard crease versus soft crease) and can be associated with the yield stress of plastic materials. The critical condition for “plastic rolling” is given by 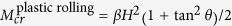. When *M* is applied, the smaller one between 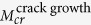 and 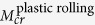 is activated as the critical moment during deformation, which leads to either “crack growth” mode, when 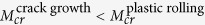, or “plastic rolling” mode, when 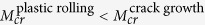.

Finite element simulations were conducted using commercial package ABAQUS to analyze these two deformation modes. Because in a LIB, Al foil tends to crack due to its lowest fracture toughness, the material parameters of Al were used in the analysis, with the surface energy *γ* 0.868 N/m, elastic modulus *E* 69 GPa, and Poisson’s ratio ν 0.33^23^,^24^. The geometry is *H* = 3 mm, and *L* = 10 mm to match with the experiments. The pre-existing crack is assumed small as compared with the width *H*. *β*, dissipated plastic energy per area, is calculated by folding a 10 μm-thick Al foil (the same thickness as that used in LIB) with different folding radius via finite element simulations. Details of this analysis are given in the Supporting Information.

Figure 3b shows the “safe zone” (i.e., 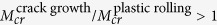) and the “fracture zone” (i.e., 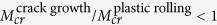) as a function of *θ* for various *a* and *β*. For example, *a* = 0.03 mm (i.e., 1% of *H*, the width of strip) and *β* = 20 MPa, corresponding in creating a sharp crease of a 10 μm-thick Al foil with bending diameter of 70 μm (see Supplementary Fig. S9), “safe mode” is activated for all angle of *θ*. The results also show that for a larger *β* (or equivalently shaper crease) or *a* (i.e., larger initial crack), “fracture mode” tends to occur. It is important to note from the Supplementary Fig. S9 that for the present battery setup (i.e., bending diameter ranging from 500 μm to 800 μm), *β* is one the order of 1 MPa, which indicates that it is always the scenario to activate the “safe mode”. Thus this analysis verifies that the robust electrochemical and mechanical performance of the kirigami LIB is due to the activated “safe mode”.

LIBs using the other two kirigami patterns were also produced and characterized. Very similar electrochemical and mechanical characteristics were exhibited during testing (Detailed results provided in Supporting Information). Supplementary Figs. S4 and S5 are for the characterizations of LIBs using the cut-N-shear and zigzag-cut patterns, respectively. The dynamic stretching deformations for LIBs using cut-N-shear and zigzag-cut patterns are shown in the Supplementary Movies S2 and S3, respectively. It is worth mentioning that the LIB using cut-N-shear pattern has doubled the energy capacity compared to the cut-N-twist pattern for a given length, and its stretchability is up to 150%.

### Connecting kirigami battery with Samsung Gear 2 smart watch

**Figure 4:**
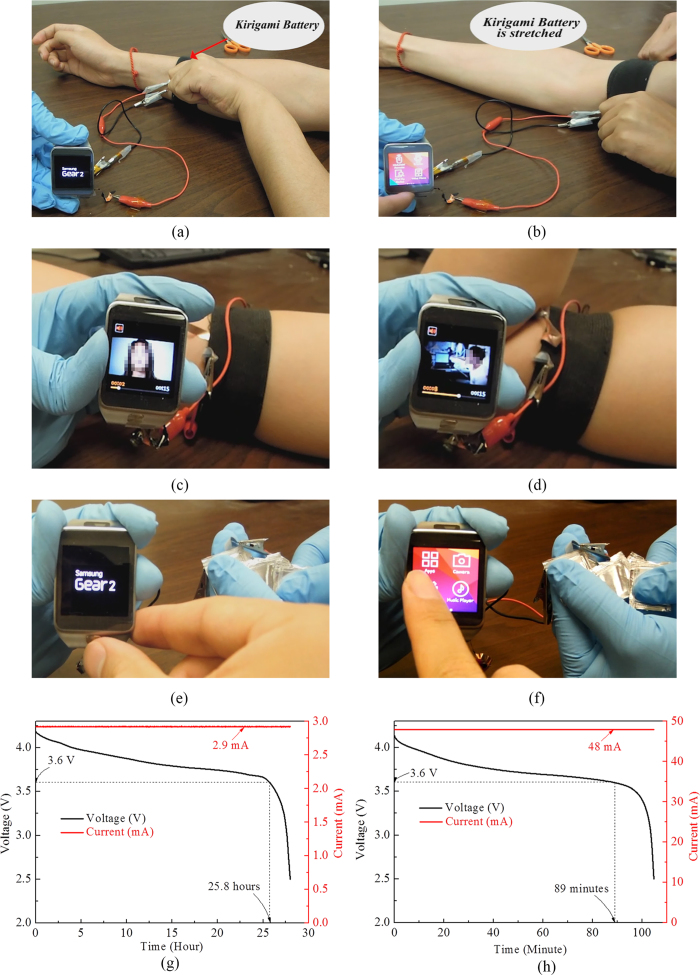
Powering a Samsung Gear 2 smart watch by a kirigami lithium-ion battery (LIB) using cut-N-twist pattern. The kirigami LIB sewn between two elastic bands was (**a**) at the wrist, (**b**) at the upper arm, (**c**) at the upper arm with elbow straightened, (**d**) at the upper arm with elbow bent. The kirigami LIB was removed from the elastic bands and stretched directly. (**e**) At the compact state and (**f**) at the stretched state. (**g**) Galvanostatic discharge to simulate the standby test with discharge current 2.9 mA. (**h**) Galvanostatic discharge to simulate the calling test with discharge current 48 mA. The stopping voltage is 3.6 V.

We demonstrated that the stretchable kirigami LIB is able to power a Samsung Gear 2 smart watch. The original LIB with energy capacity 300 mAh was removed from the Samsung Gear 2 and a kirigami LIB using cut-N-twist pattern was connected to the device. The same geometry as that in Fig. 2 was used. The mass loading for active materials are 0.26 g for graphite, 0.65 g for LCO, which gives the energy capacity 80 mAh. At the compact state, the kirigami LIB is 51.3 mm in length, 27 mm in width, and 2.6 mm in thickness. The produced kirigami LIB was sewn between two elastic bands at its two ends and wrapped around the wrist, allowing the elastic bands to function as an elastic watch strap. By sewing the kirigami LIB to the elastic band at the two ends, the LIB can be stretched and contracted, driven by the elasticity of the band. The Supplementary Movie S4 shows a series dynamic deformation on the elastic “watch strap” and thus on the kirigami LIB, and some snapshots are provided in Fig. 4. Figure 4a shows that when the elastic band and the kirigami LIB were at their most compact states, the Samsung Gear 2 was just turned on. Then, while the elastic band was stretched from the wrist to the upper arm, the Samsung Gear 2 was working normally (Fig. 4b). It is estimated from the circumferences of the wrist and upper arm, the kirigami LIB was subjected to a strain of 30%, lower than its full stretchability. While the elbow bent (Fig. 4c) and straightened (Fig. 4d), the smart watch was able to maintain normal functionality, and even display a video. During bending and straightening of the elbow, the biceps introduced an additional 15% strain to the kirigami LIB. Finally, the kirigami LIB was removed from the elastic bands and stretched directly while powering the smart watch (Fig. 4e,f).

To further evaluate the performance of the kirigami battery, standby and calling tests of the smart watch powered by the kirigami battery were carried out. For a fully charged kirigami battery with 80 mAh capacity that was connected with a Samsung Gear 2 smart watch, the standby time was measured to be 24.5 hours. When the smart watch was paired with a Samsung Galaxy S5 cell phone with a Bluetooth connection when they were separated by 30 cm, the smart watch was able to make calls through the Bluetooth connection. The calling time was measured to be 90 minutes. To simulate the standby and calling tests, quantitative discharge characterizations were also conducted by applying the corresponding constant discharge currents for standby (2.9 mA) and calling (48 mA) using an Arbin electrochemical workstation. The stopping voltage of the smart watch was measured to be 3.6 V. Details of the measurement of the discharge currents and stopping voltage are provided in the Supplementary Information. As shown in Fig. 4g for the simulated standby test, the calling time (when the voltage drops to the stopping voltage 3.6 V) is 25.8 hours, which is consistent with the direct test. For the simulated calling test (Fig. 4h), the calling time is 90 minutes, which perfectly matches the direct test. It should be noticed that the discharge currents for standby and calling are relatively low C-rate for the present kirigami battery with 80 mAh capacity, specifically discharge current for standby 2.9 mA ≈ C/30 and that for calling 48 mA ≈ C/2. Given the stable rate performance as shown in Fig. 2d (though for a kirigami battery with different capacity), a stable cyclic charge/discharge performance can be expected. These experiments demonstrate the promises of using a kirigami LIB to replace present rigid and bulky batteries and to power a commercial smart watch, which has been a bottle-neck in developing compact wearable devices. It is worth mentioning that if the kirigami LIB is scaled up to cover the entire area of the elastic band (25 cm in length, 3 cm in width) with a battery thickness of 0.3 cm, the energy capacity is about 700 mAh, which significantly exceeds the current LIB used in most smart watches, and the energy density is about 160 Wh/Kg, which is comparable to the current LIB used in smart watches or smart phones. By using the space of watch strap instead of using the limited space of watch body, the kirigami battery may disruptively impact the field of wearable electronics by offering extra physical and functional design space. Furthermore, to test the compatibility of the Kirigami battery integrated with real watchband, a Cut-N-Twist battery was embedded in a watchband made of Sorta-Clear 40 (Smooth-On, Inc.). Movie S5 shows the watchband integrated with Kirigami battery powering the Samsung Gear 2.

### Thermal test of kirigami battery and Samsung Gear 2 battery

The Kirigami battery is thin film based. It is has much higher surface-to-volume ratio than bulky battery, which is beneficial for heat radiation. The thermal test of Kirigami battery and Samsung Gear 2 bulky battery were conducted. Figure S10 and S11 illustrates the test and the result. In the test both batteries discharged at 48 mA for one hour. Obvious temperature rise was observed for the Gear 2 bulky battery, while the Kirigami battery was consistent with the ambient temperature during the discharge period.

## Discussion

The demonstration of stretchable kirigami LIBs in a Samsung smart watch only represents one application of this type of stretchable energy sources that fully utilize the mainstream manufacturing capability. Other applications may include smart bracelets and smart headbands among many others. It is expected that the kirigami LIBs are able to resolve one of the bottlenecks in the development of wearable devices by providing a scalable solution for a stretchable energy source to profoundly change the form factor. The methodology involved in kirigami-based approach, i.e., competing mechanisms between “crack growth” and “plastic rolling” also provide a much broader spectrum of employing the concept of kirigami to other fields, such as in microelectromechanical systems (MEMS) where robust interconnects can be placed at the cut/fold locations and the functional devices are fabricated on the rigid faces, which leads to stretchable devices using standardized procedures. These areas appear promising for further research.

## Methods

### Fabrication of kirigami lithium-ion batteries

The multilayer stacking structures as shown in Supplementary Fig. S2 were used to fabricate the lithium-ion batteries (LIBs), where graphite (Fisher Scientific Inc.) and LiCoO2 (LCO, MTI Corp. ) as active materials for anode and cathode electrodes, respectively; copper (Cu) and aluminum (Al) as the anode and cathode current collectors, respectively; polypropylene (Celgard 2500) as separator, 1 M LiPF6 in EC:DMC:DEC (1:1:1) as electrolyte, and the aluminized polyethylene (PE) (Sigma-Aldrich) was the packaging material. Cathode slurries were prepared by mixing the LCO, PVDF (MTI Corp.), Carbon black (Super C45) and N-Methyl-2-pyrrolidone solvent (CreoSalus) with a ratio of 8:1:1:5 by weight. Then the slurry was uniformly coated on a 10 μm-thick Al foil (Reynolds Wrap), and then dried on a hot plate at 130 ^°^C for 5 hours in ambient environment. Anode slurries were prepared by mixing the graphite (Fisher Scientific), carbon black (Super C45), Carboxymethyl cellulose (Fisher Scientific), Styrene Butadiene Rubber (Fisher Scientific) and DI water with a ratio of 95:2.5:1.25:1.25:200 by weight. After that the slurry was uniformly coated on a 20 μm-thick Cu foil (CF-T8G-UN, Pred Materials International, Inc.), and then dried on a hot plate at 120 ^°^C for 5 hours in ambient environment. A mass ratio for graphite:LCO was around 1:2.5. Finally the anode and cathode electrodes were pressed to make condensed electrodes. The multilayer structure shown in Supplementary Fig. S2 was subjected to folding and cutting following the kirigami patterns given by Supplementary Fig. S1.

### Electrochemical characterization

An Arbin electrochemical workstation with a cutoff voltage of 2.5–4.2 V at room temperature was used to conduct cyclic galvanostatic charge and discharge of the kirigami batteries at the most compact and the stretched states. The maximum output power of the fully charged battery was calculated by 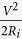, where *V* is the open circuit voltage and *R*_*i*_ is the internal resistance as a function of system-level mechanical strain and cycles of mechanical loading. The values of voltage of the kirigami batteries were measured using a voltmeter. The electrochemical impedance spectroscopy (EIS) characterizations were performed by applying a small perturbation voltage of 5 mV in the frequency range of 0.1 Hz to 100 kHz during the first discharge cycle before and after stretching, using a Gamry Echem Analyst. The analysis of the impedance spectra was conducted using equivalent circuit software provided by the manufacturer.

### Measurement of the discharge currents for standby and calling of the Samsung Gear 2 watch

The discharge current for standby was measured to be about 2.9 mA when the smart watch was turned on and remained at the standby mode as the kirigami battery was connected. To measure the discharge current for calling, the Samsung Gear 2 watch was paired with a Samsung Galaxy S5 cell phone with a Bluetooth connection when they were separated by 30 cm. A phone call was made via the cell phone and accepted from the smart watch end. The discharge current for calling was then measured to be about 48 mA.

### Measurement of the stopping voltage of the Samsung Gear 2 watch

After the smart watch stopped working, the kirigami battery was disconnected from the watch. The voltage leftover in the battery was then measured. The stopping voltages of both standby and calling test were measured to be about 3.6 V.

### Measurement of the Kirigami battery and Samsung Gear 2 battery temperature

The temperature was measured by thermal couple. Two thermal couples were used during one measurement. One was attached to the battery. The other was attached to the testing table (ambient). Data was collected every 3 miniutes.

## Additional Information

**How to cite this article**: Song, Z. *et al.* Kirigami-based stretchable lithium-ion batteries. *Sci. Rep.* **5**, 10988; doi: 10.1038/srep10988 (2015).

## Supplementary Material

Supplementary Information

Supplementary Movie S1

Supplementary Movie S2

Supplementary Movie S3

Supplementary Movie S4

Supplementary Movie S5
