# Supplementary material for: Kirigami-based stretchable lithium-ion batteries
Source: Sci Rep. 2015 Jun 11;5:10988. doi: 10.1038/srep10988 (PMC4463940; doi:10.1038/srep10988)
Supplement: Supplementary Information [file srep10988-s1.pdf]

Supporting Information

for

Kirigami based stretchable lithium-ion batteries

Zeming Song[1#], Xu Wang[1#], Cheng Lv[1], Yonghao An[1], Mengbing Liang[2], Teng Ma[1], David He[1,2], Ying-Jie Zheng[3], Shi-Qing Huang[3], Hongyu Yu[4,5], and Hanqing Jiang[1,*]

[1]School for Engineering of Matter, Transport and Energy, Arizona State University, Tempe, AZ 85287, USA

[2]Desert Vista High School, Phoenix, AZ 85048, USA

[3]MOE Key Lab of Disaster Forecast and Control in Engineering, Jinan University, Guangzhou 510632, China

[4]School of Electrical, Computer and Energy Engineering, Arizona State University, Tempe, AZ 85287, USA

[5]School of Earth and Space Exploration, Arizona State University, Tempe, AZ 85287, USA

#These authors contribute equally.

*Email: hanqing.jiang@asu.edu

Geometry of the Three Kirigami Patterns for Lithium-Ion Batteries (LIBs)

In order to ensure the quality of the packaging, specifically, no short between anode and cathode, and large areal coverage, the following geometries were used to produce the three kirigami batteries with these three patterns, namely, zigzag-cut, cut-N-twist, and cut-N-shear patterns. The annotation shown in the Fig. S1 is the size of the current collector with electrode materials. For the LIB stack, the size of the separator is 1mm larger than that of the current collector at each edge to ensure the separation of the two electrodes and the size of the package materials is 2 mm even larger than that of the separator at each edge to ensure the film sealing of the battery.

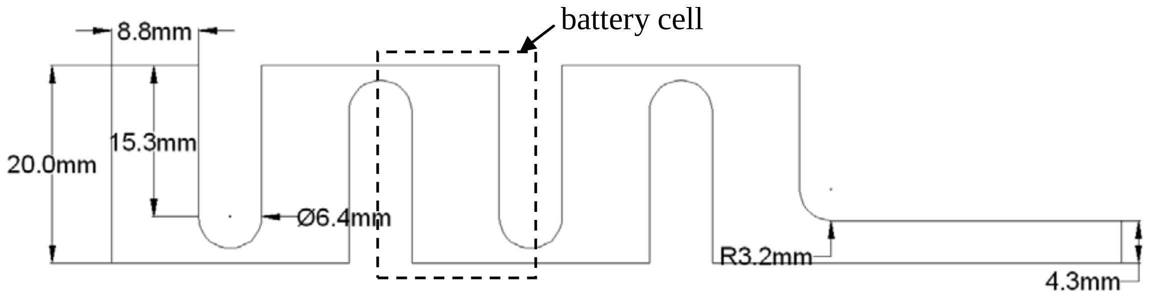

(a)

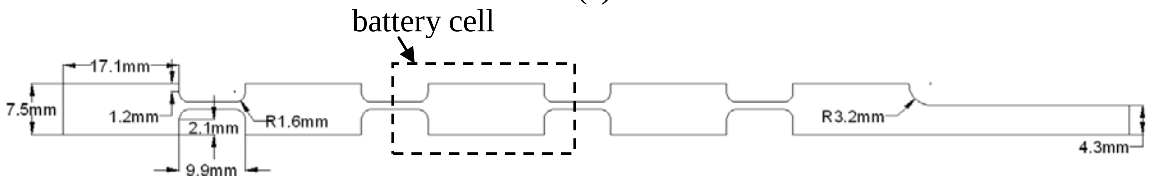

(b)

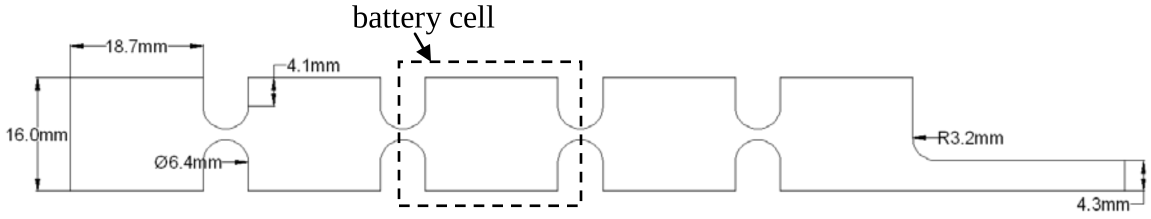

(c)

Supplementary Figure S1. Geometries of the three kirigami batteries. (a) Zigzag-cut pattern, **(b)** cut-N-twist pattern, and **(c)** cut-N-shear pattern. The dashed line shows a battery cell.

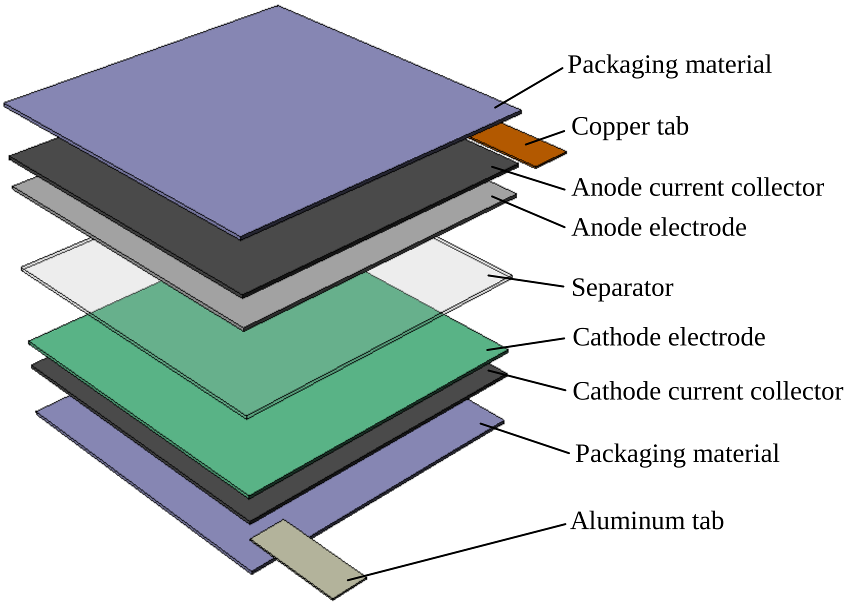

Supplementary Figure S2. Exploded view of the multilayer structure of lithium-ion batteries.

Lighting up a Light-Emitting Diodes (LED) by a Kirigami Lithium-Ion Battery (LIB) using Cut-N-Twist Pattern

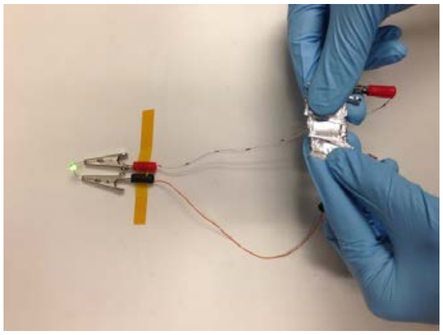

(a)

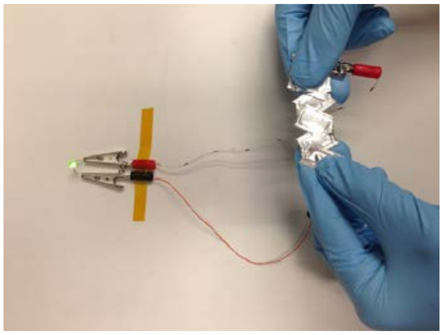

(b)

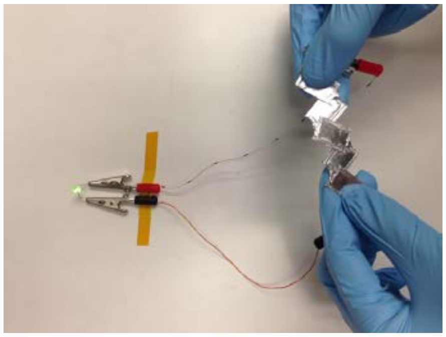

(c)

Supplementary Figure S3. A kirigami lithium-ion battery (LIB) using cut-N-twist pattern is lighting up a light-emitting diodes (LED) while (a) at the most compact state, **(b)** being stretched by 30%, and **(c)** being stretched by 70%.

Electrochemical and Mechanical Characterization of a Kirigami Lithium-Ion Battery using Cut-N-Shear Pattern

Similar to Fig. 2 in the main text, the kirigami lithium-ion batteries (LIBs) were produced by following the geometry given by Supplementary Fig. S1c. Then the electrochemical and mechanical characterizations were performed.

Figure S4 shows electrochemical and mechanical characterization results for LIBs using cut-N-shear pattern. Figures S4a and S4b show the images the LIB at the most compact and stretched states. Fig. S4b shows that a LIB using cut-N-shear pattern can be stretched up to 150% by using the most compact state (Fig. S4a) as the reference. Meanwhile the thickness change, from 4.30 mm at the most compact state to 2.7 mm at the most stretched state is noticeable. Figure S4c shows the electrochemical cycling results of a LIB using the cut-N-shear pattern at its most compact state (for the 1st to 5th cycles), followed by that at its most stretched state (for the 6th to 10th cycles), then that at its most compact state again (for the 11th to 15th cycles) and finally followed by that at its most stretched state again (for the 16th to 20th cycles) under C/3 charge/discharge rate. Fairly stable charge/discharge behaviors under the compact and stretched states are observed. The present mass loading (see caption of Fig. S4c) gives this kirigami LIB 75 mAh energy capacity. Figure S4d shows the cyclic stability of the LIBs up to 20 cycles. Figure S4e shows the excellent rate performance of this kirigami battery when the charge/discharge rate varied in the sequence of C/3, C/2, C and C/3 again at both compact and stretched state. Figure S4f provides the results for electrochemical impedance spectroscopy (EIS) studies during the first discharge cycle at the most compact state before stretching and stretched state after 100 cycles of stretching. No significant changes in the impedance were found.

Then the mechanical characteristics of the fully charged kirigami LIB using cut-N-shear are examined. As shown in Fig. S4g, at different stretchability, the output voltage remained steady at 3.87 V. Supplementary Movie S2 shows the dynamic process of this deformation. Figure S4h shows the maximum output power of the kirigami LIB as a function of stretchability, $\varepsilon_{stretch}$, under different cycles of stretching. The internal resistance of the battery is measured to be about 1.5 Ω. Over 3,000 stretching cycles and up to a stretchability $\varepsilon_{stretch}$ of 83 %, there is insignificant decrease of the power. The maximum output power is 4.7 W and is sufficient to operate commercial light-emitting diodes (LEDs). As shown in the Supplementary Movie S2, LEDs driven by this kirigami LIB do not show noticeable dimming upon cyclic stretching. Ultimate tensile strength of LIBs using cut-N-shear pattern 11.5 MPa with load frame Instron-Model 4411.

Figures S4i and S4j show the scanning electron micrographs (SEMs) for the anode current collectors (e.g., Cu foil) at the cuts before charging, and after discharge and 100 cycles of mechanical deformation. The similar SEM images are given for the cathode current collectors (e.g., Al foils) in Figs. S4k and S4l. There are no cracks after cyclic mechanical stretching.

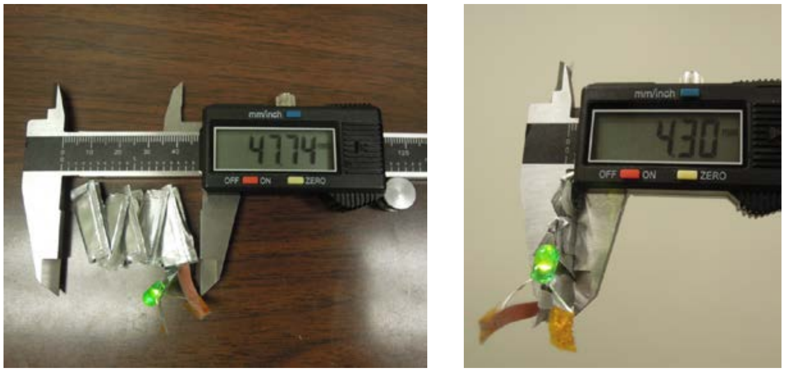

(a)

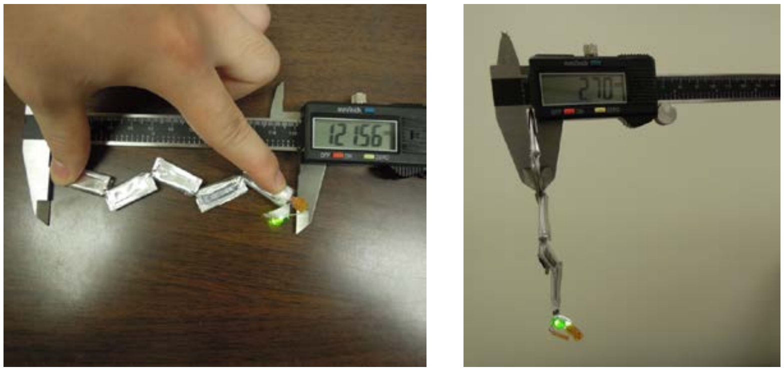

(b)

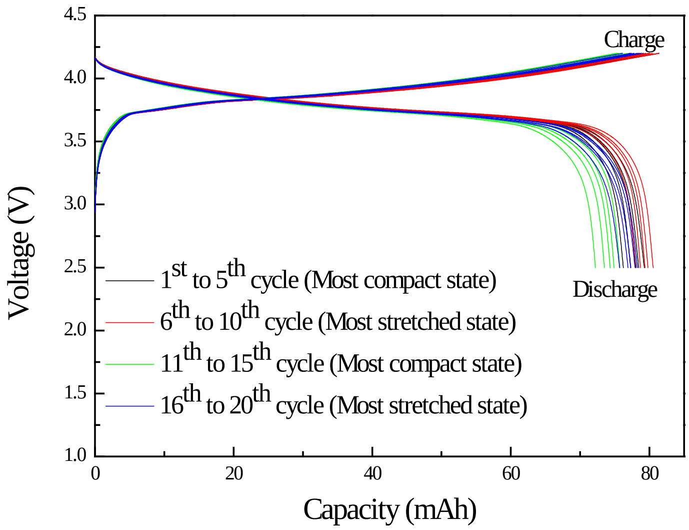
Voltage (V)
Capacity (mAh)
Charge
Discharge
1st to 5th cycle (Most compact state)
6th to 10th cycle (Most stretched state)
11th to 15th cycle (Most compact state)
16th to 20th cycle (Most stretched state)
4.5
4.0
3.5
3.0
2.5
2.0
1.5
1.0
0
20
40
60
80

(c)

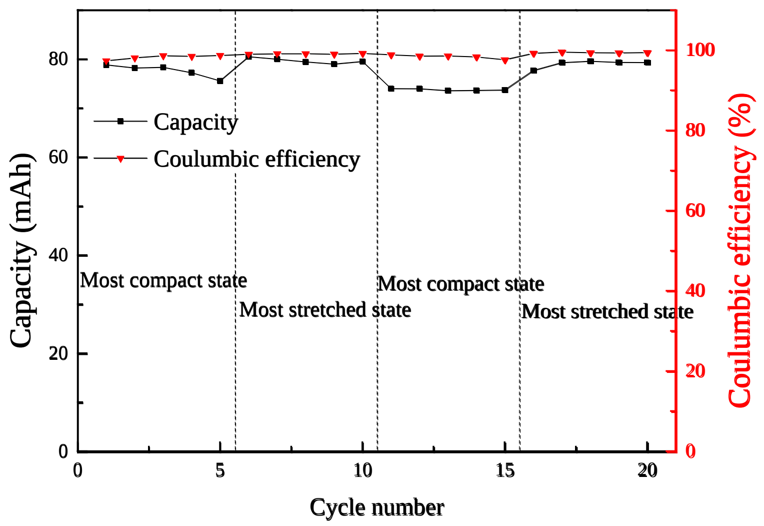
Capacity
Coulumbic efficiency
Most compact state
Most stretched state
Most compact state
Most stretched state
Capacity (mAh)
Coulumbic efficiency (%)
Cycle number
0
20
40
60
80
100
0
5
10
15
20

(d)

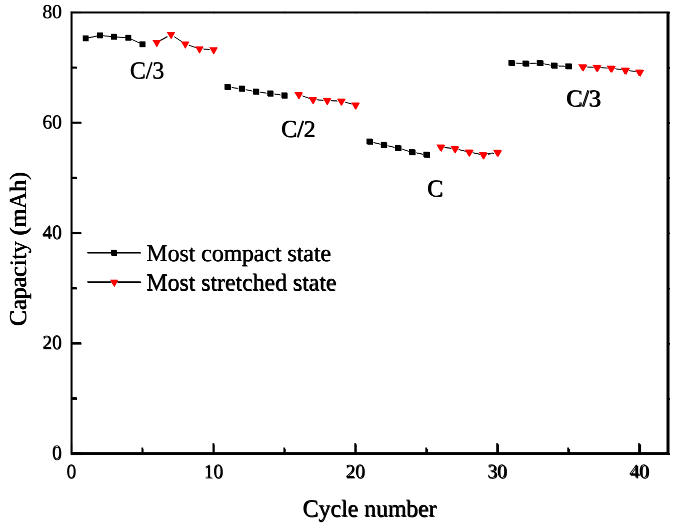
C/3
C/2
C
C/3
Most compact state
Most stretched state
Capacity (mAh)
Cycle number
0
20
40
60
80
0
10
20
30
40

(e)

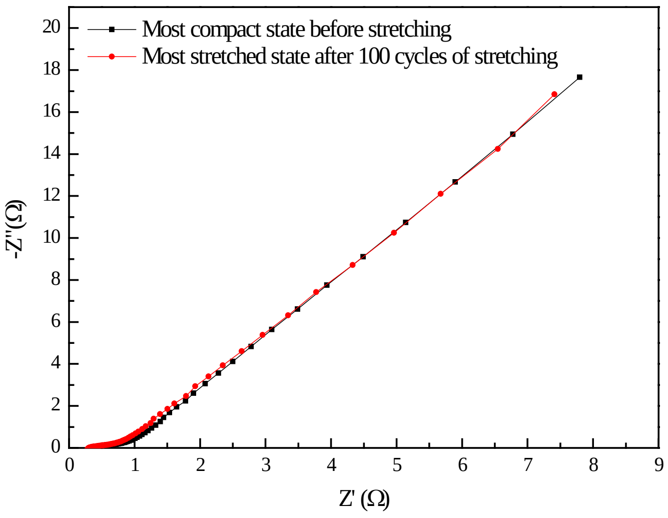
Most compact state before stretching
Most stretched state after 100 cycles of stretching
-Z''(Ω)
Z' (Ω)
0
2
4
6
8
10
12
14
16
18
20
0
1
2
3
4
5
6
7
8
9

(f)

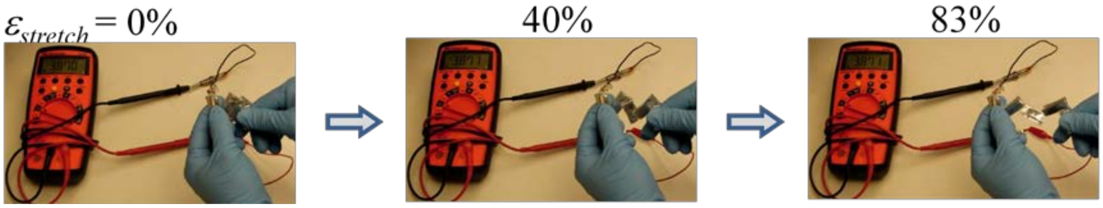
$\varepsilon_{stretch}$ = 0%
40%
83%

(g)

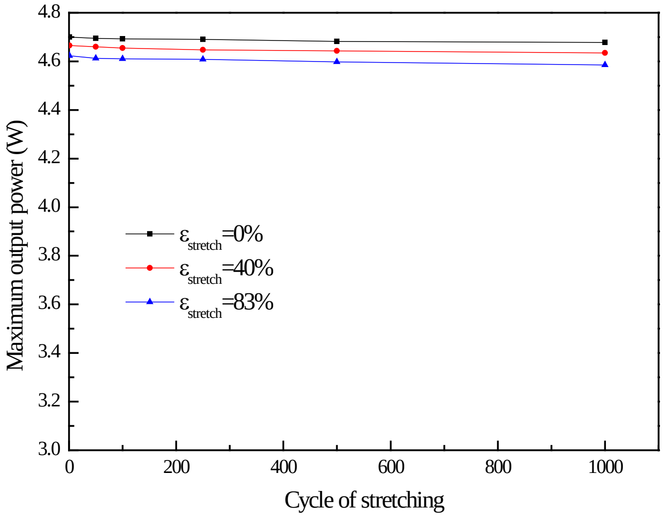
Maximum output power (W)
Cycle of stretching
$\varepsilon_{stretch}$=0%
$\varepsilon_{stretch}$=40%
$\varepsilon_{stretch}$=83%
3.0
3.2
3.4
3.6
3.8
4.0
4.2
4.4
4.6
4.8
0
200
400
600
800
1000

(h)

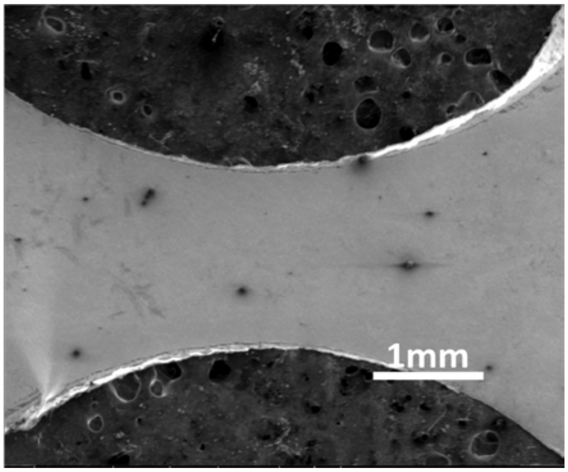

(i)

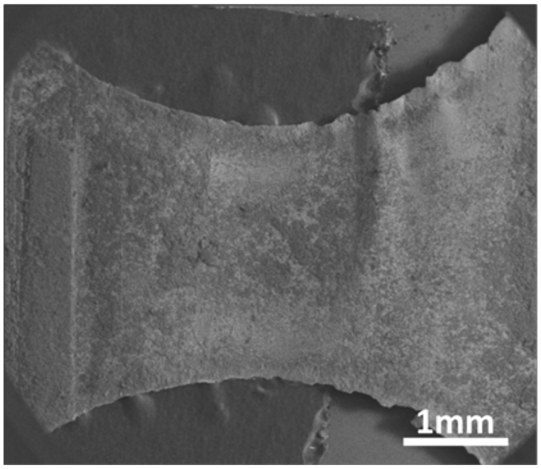

(j)

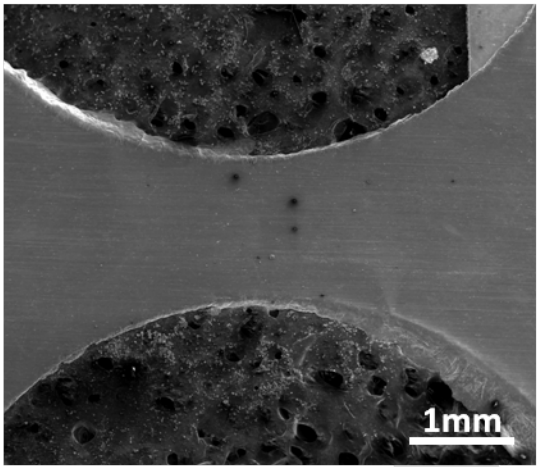

(k)

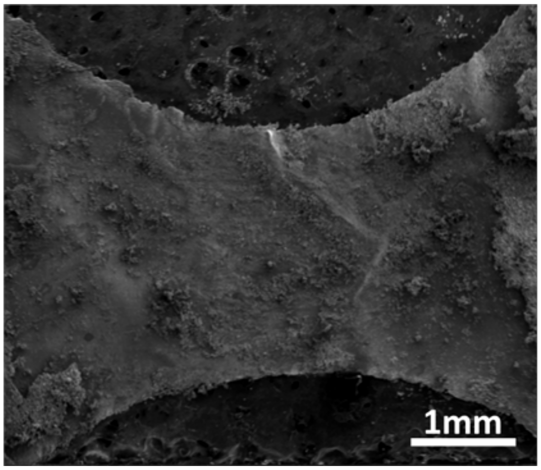

(l)

Supplementary Figure S4. Electrochemical and mechanical characterization of a kirigami lithium-ion battery (LIB) using cut-N-shear pattern. (a) Photograph of a LIB at its most compact state. **(b)** Photograph of a LIB at its most stretched state. **(c)** Galvanostatic charge and discharge at the most compact state (1st to 5th cycles), the most stretched state (6th to 10th cycles), the most compact state again (11th to 15th cycles), and the most stretched state again (16th to 20th cycles) under C/3 charge/discharge rate. The mass loading of $LiCoO_2$ (LCO) (specific capacity 145 mAh g^{-1}) and graphite (specific capacity 372 mAh g^{-1}) were 240 mg and 650 mg, respectively, which gave LIB the capacity of 75 mAh. **(d)** Energy capacity (left axis, black) and Coulombic efficiency (right axis, red) as a function of cycle number for C/3 charge/discharge rate. The mass accounts for all the materials involved in a cell, which is 2.98 g. **(e)** Rate performance when the charge/discharge rates varied from C/3, C/2, to C, and C/3 again for both compact and stretched states. When discharge rates increase, as expected, the capacity decreases from 73.2 mAh for C/3 rate to 66.5 mAh for C/2 rate, and 56.6 mAh for discharge rate C. However, the capacity recovered to the 70.8 mAh when the discharge rate resumed to C/3 after 30 cycles charge/discharge at the both compact the stretched state under varies C-rates, which indicates excellent rate performance of this kirigami battery. **(f)** Electrochemical

impedance spectroscopy (EIS) analysis during the first discharge cycle at the most compact state before stretching and stretched state after 100 stretching cycles. EIS studies were performed by applying a small perturbation voltage of 5 mV in the frequency range of 0.1 Hz to 100 kHz. Typical impedance spectrum, with high-to-middle frequency range flat curve and a relative straight line representing the low frequency range, was observed. No obvious semicircle was observed because of the low internal resistant. There are not significant changes in the impedance before and after mechanical deformation. **(g)** Photograph of stretching a kirigami LIB while it was connected to a voltmeter. **(h)** Maximum output power of the kirigami LIB as a function of stretchability over 3,000 cycles of stretches. **(i)** Scanning electron micrographs (SEM) of anode current collector Cu at the cut before charge. **(j)** SEM of anode current collector Cu at the cut after discharge and 100 stretching. **(k)** SEM of cathode current collector Al at the cut before charge. **(l)** SEM of cathode current collector Al at the cut after discharge and 100 stretching.

Electrochemical and Mechanical Characterization of a Kirigami Lithium-Ion Battery using Zigzag-Cut Pattern

Similar to Fig. 2 in the main text, the kirigami lithium-ion batteries (LIBs) was produced by following the geometry given by Supplementary Fig. S1a. Then the electrochemical and mechanical characterizations were performed.

Figure S5 shows electrochemical and mechanical characterization results for LIBs using zigzag-cut pattern. Figures S5a and S5b show the pictures of the LIB at the most compact and stretched states. The LIB using zigzag-cut pattern has relatively small stretchability, approximately 46% measured from the most compact state (Fig. S5a) to the most stretched state (Fig. S5b), and out-of-plane deformation can be observed when stretched (Fig. S5b). Similar cyclic charge/discharge curves are shown in Fig. S5c and S5d. The present mass loading gives this kirigami LIB 55 mAh energy capacity. The rate performance of this kirigami battery when the charge/discharge rate varied in the sequence of C/3, C/2, C and C/3 again at both compact and stretched state was given by Fig. S5e. Electrochemical impedance spectroscopy (EIS) studies during the first discharge cycle at the most compact state before stretching and stretched state after 100 cycles of stretching are shown in Fig. S5f. No significant changes in the impedance were found.

The mechanical characteristics of the fully charged kirigami LIB using zigzag-Cut are then examined. Figure S5g shows that at different stretchability, the output voltage remained steady at 3.86 V. Supplementary Movie S3 shows the dynamic process of this deformation. Figure S5h shows the maximum output power of the kirigami LIB as a function of stretchability, $\varepsilon_{stretch}$, under different cycles of stretching. Here the internal resistance of the battery is measured to be

about 1.7 Ω. Over 3,000 stretching cycles and up to a stretchability $\varepsilon_{stretch}$ of 35%, there is no obvious output power decay. The output power of 4.7 W is sufficient to operate commercial light-emitting diodes (LEDs). As shown in Supplementary Movie S3, LEDs driven by this kirigami LIB do not show noticeable dimming upon cyclic stretching. Ultimate tensile strength of LIBs using zigzag-cut pattern is 10.8 MPa with load frame Instron-Model 4411.

Figures S5i and S5j show the scanning electron micrographs (SEMs) for the anode current collectors (e.g., Cu foil) at the cuts before charging, and after discharge and 100 cycles mechanical deformation. The similar SEM images are given for the cathode current collectors (e.g., Al foils) in Figs. S5k and S5l. Again, no cracks are observed after cyclic mechanical stretching.

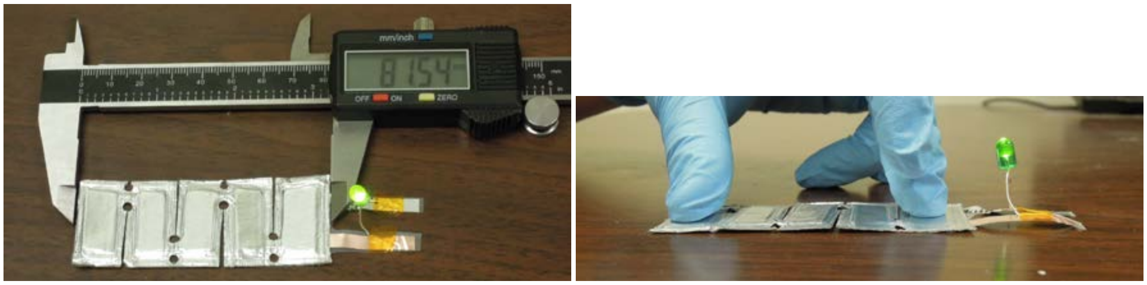

(a)

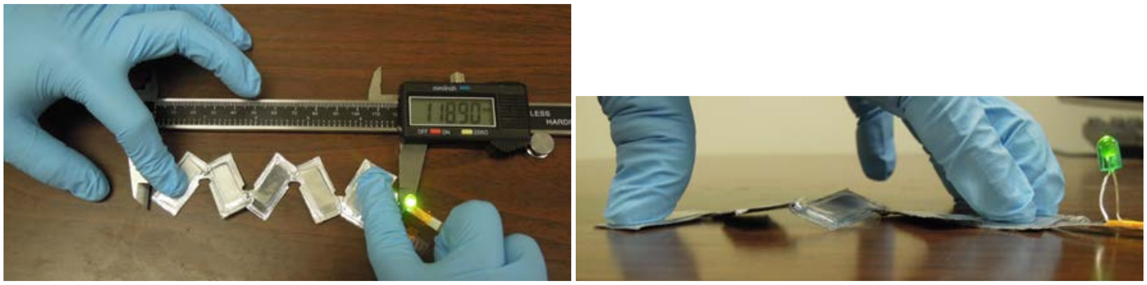

(b)

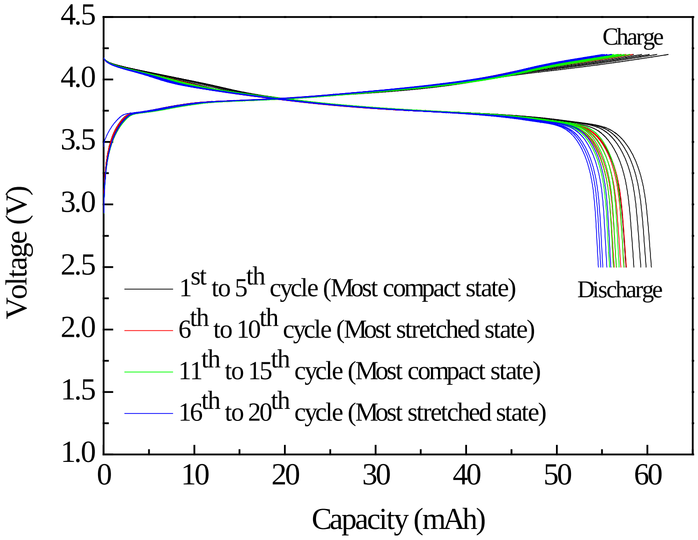

Charge
Discharge
Voltage (V)
Capacity (mAh)
4.5
4.0
3.5
3.0
2.5
2.0
1.5
1.0
0
10
20
30
40
50
60
1st to 5th cycle (Most compact state)
6th to 10th cycle (Most stretched state)
11th to 15th cycle (Most compact state)
16th to 20th cycle (Most stretched state)

(c)

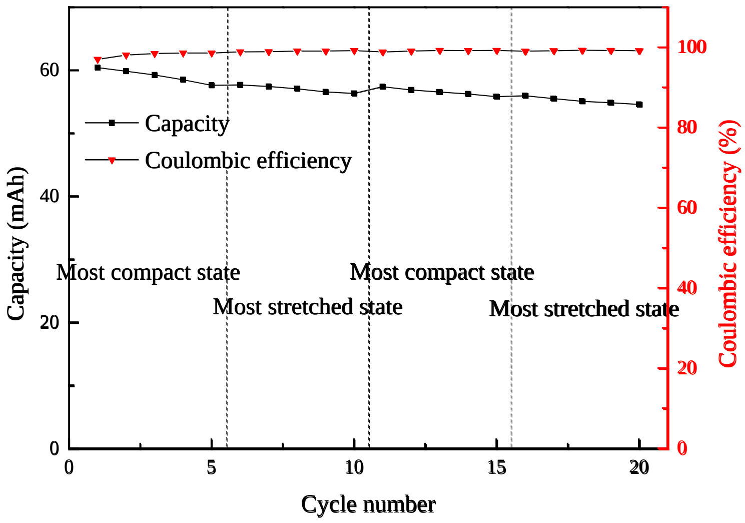
Capacity
Coulombic efficiency
Most compact state
Most stretched state
Most compact state
Most stretched state
Capacity (mAh)
Coulombic efficiency (%)
Cycle number
0
20
40
60
0
20
40
60
80
100
0
5
10
15
20

(d)

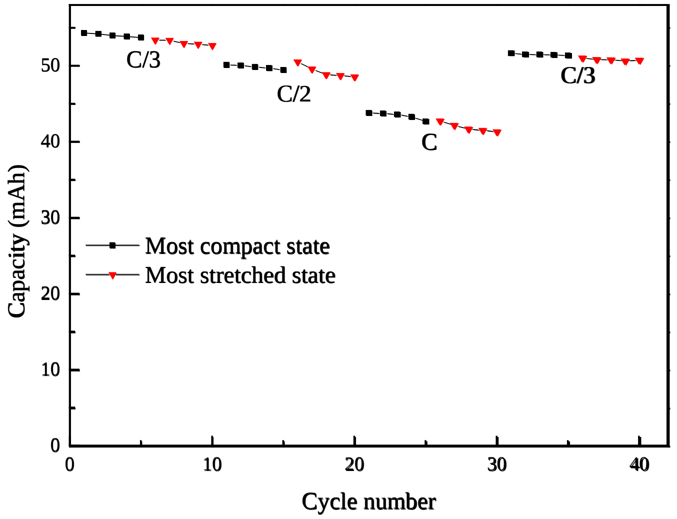
C/3
C/2
C
C/3
Most compact state
Most stretched state
Capacity (mAh)
Cycle number
0
10
20
30
40
50
0
10
20
30
40

(e)

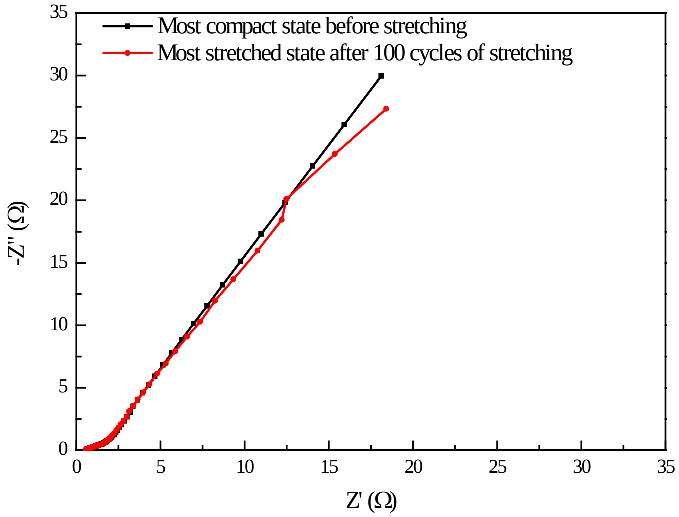
Most compact state before stretching
Most stretched state after 100 cycles of stretching
-Z'' (Ω)
Z' (Ω)
0
5
10
15
20
25
30
35

(f)

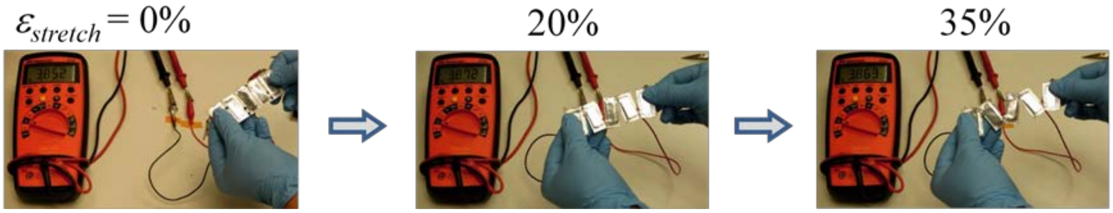
$\varepsilon_{stretch}$ = 0%
20%
35%

(g)

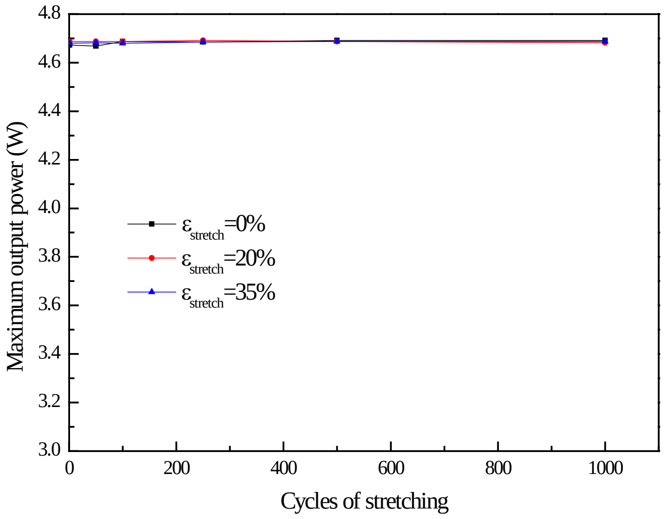
$\varepsilon_{stretch}$=0%
$\varepsilon_{stretch}$=20%
$\varepsilon_{stretch}$=35%
Maximum output power (W)
Cycles of stretching
3.0
3.2
3.4
3.6
3.8
4.0
4.2
4.4
4.6
4.8
0
200
400
600
800
1000

(h)

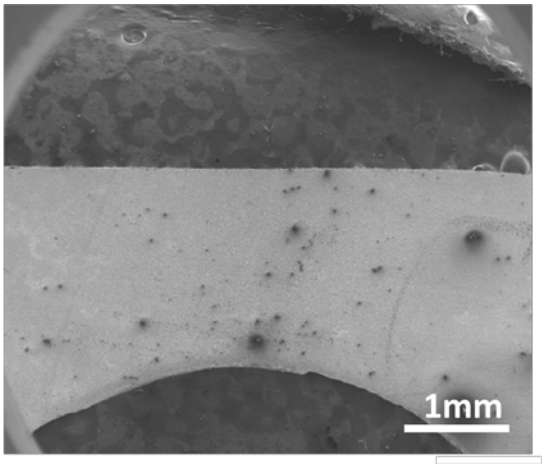

(i)

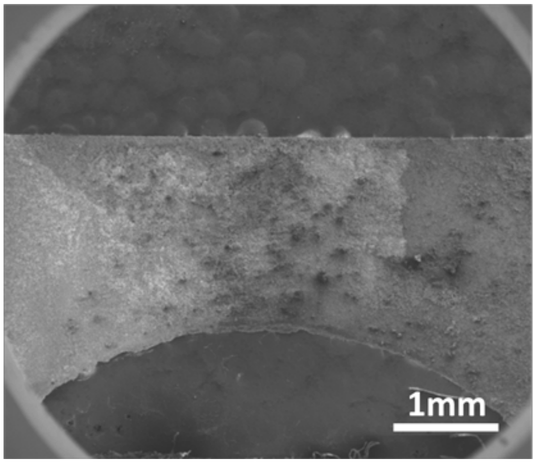

(j)

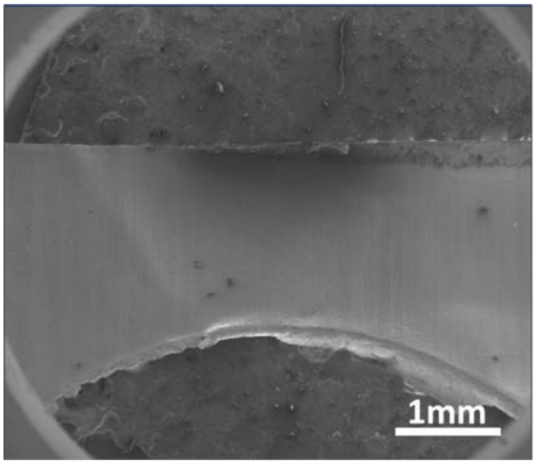

(k)

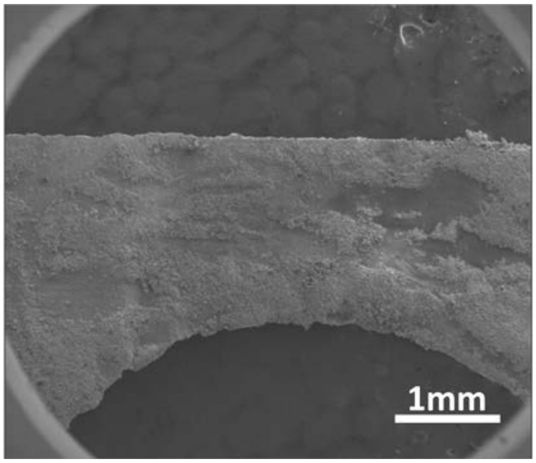

(l)

Supplementary Figure S5. Electrochemical and mechanical characterization of a kirigami lithium-ion battery (LIB) using zigzag-cut pattern. (a) Photograph of a LIB at its most compact state. **(b)** Photograph of a LIB at its most stretched state. **(c)** Galvanostatic charge and discharge at the most compact state (1st to 5th cycles), the most stretched state (6th to 10th cycles), the most compact state again (11th to 15th cycles), and the most stretched state again (16th to 20th cycles) under C/3 charge/discharge rate. The mass loading of $LiCoO_2$ (LCO) (specific capacity 145 mAh g^{-1}) and graphite (specific capacity 372 mAh g^{-1}) were 174 mg and 457 mg, respectively, which gave LIB the capacity of 55 mAh. **(d)** Energy capacity (left axis, black) and Coulombic efficiency (right axis, red) as a function of cycle number for C/3 charge/discharge rate. The mass accounts for all the materials involved in a cell, which is 2.10 g. **(e)** Rate performance when the charge/discharge rates varied from C/3, C/2, to C, and C/3 again for both compact and stretched states. When discharge rates increase, as expected, the capacity decreases from 52.7 mAh for C/3 rate to 50.1 mAh for C/2 rate, and 43.8 mAh for discharge rate C. However, the capacity recovered to the 51.7 mAh when the discharge rate resumed to C/3 after 30 cycles charge/discharge at the both compact the stretched state under varies C-rates, which indicates excellent rate performance of this kirigami battery. **(f)** Electrochemical

impedance spectroscopy (EIS) analysis during the first discharge cycle at the most compact state before stretching and stretched state after 100 stretching cycles. EIS studies were performed by applying a small perturbation voltage of 5 mV in the frequency range of 0.1 Hz to 100 kHz. Typical impedance spectrum, with high-to-middle frequency range flat curve and a relative straight line representing the low frequency range, was observed. No obvious semicircle was observed because of the low internal resistant. There are not significant changes in the impedance before and after mechanical deformation. **(g)** Photograph of stretching a kirigami LIB while it was connected to a voltmeter. **(h)** Maximum output power of the kirigami LIB as a function of stretchability over 3,000 cycles of stretches. **(i)** Scanning electron micrographs (SEM) of anode current collector Cu at the cut before charge. **(j)** SEM of anode current collector Cu at the cut after discharge and 100 stretching. **(k)** SEM of cathode current collector Al at the cut before charge. **(l)** SEM of cathode current collector Al at the cut after discharge and 100 stretching.

Theoretical Analysis of the Two Competing Mechanisms, "Crack Growth" versus "Plastic Rolling"

According to the Griffith’s criterion for linear elastic fracture, potential energy takes this form , $\Pi^{\text{crack growth}} \sim \sigma^2 a^2 / E$, where σ is the normal stress on the crack, a is the size of the crack and E is the elastic modulus. The energy releasing rate due to the crack growth is then given by $J = \partial \Pi^{\text{crack growth}} / \partial a \sim \sigma^2 a / E$. Here J is the J-integral which equals to the energy release per unit area. For the present scenario that has different geometrical setup as that in the Griffith's criterion, the geometry factors are taken into account. The form of J is speculated to be $A\sigma^2 a / E$, where A characterizes the geometrical effects and is determined by finite element simulations. A is assumed to be a function of θ .

In finite element simulations using commercial package ABAQUS, the values of σ , a and E are fixed while θ changes from 0 to $\pi / 3$. Plane stress model was applied as the structure has very low thickness compared to its in-plane dimensions. Mesh was refined around the crack tip, which is shown in Supplementary Fig. S6 for $\theta = \pi / 4$. About 200,000 CPS4R (4-node bilinear plane stress quadrilateral, reduced integration) elements were used to obtain accurate and converged results. *J*-integral was obtained by numerical integration along the elements on a circle with the crack tip as its center. After obtaining the values of *J*-integral for different angle θ , the value of A can be calculated by the expression of J as mentioned

above, i.e. $J = A\sigma^2 a / E$. Substitute A into the expression of $M_{cr}^{\text{crack growth}}$ and the final expression of $M_{cr}^{\text{crack growth}}$ is thus obtained.

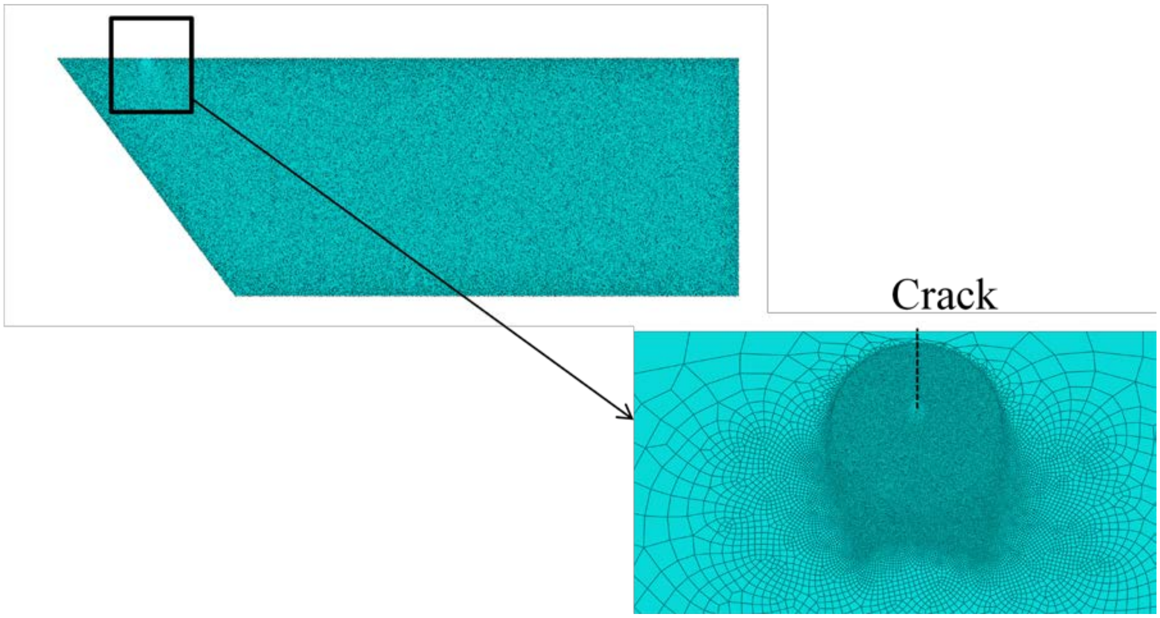

Supplementary Figure S6. Mesh of finite element model for $\theta = \pi / 4$.

During the plastic rolling (i.e., the angle θ changes), the plastic zone is highlighted by the shaded area as shown in Supplementary Fig. S7. The area of the plastic zone is $H^2 \tan\theta / 4$. So the critical moment $M_{cr}^{\text{plastic rolling}}$ for plastic rolling can be obtained by $M_{cr}^{\text{plastic rolling}} = \partial \Pi^{\text{plastic rolling}} / \partial\theta = \partial\left(\beta H^2 \tan\theta / 4\right) / \partial\theta = \beta H^2 \left(1 + \tan^2\theta\right) / 2$.

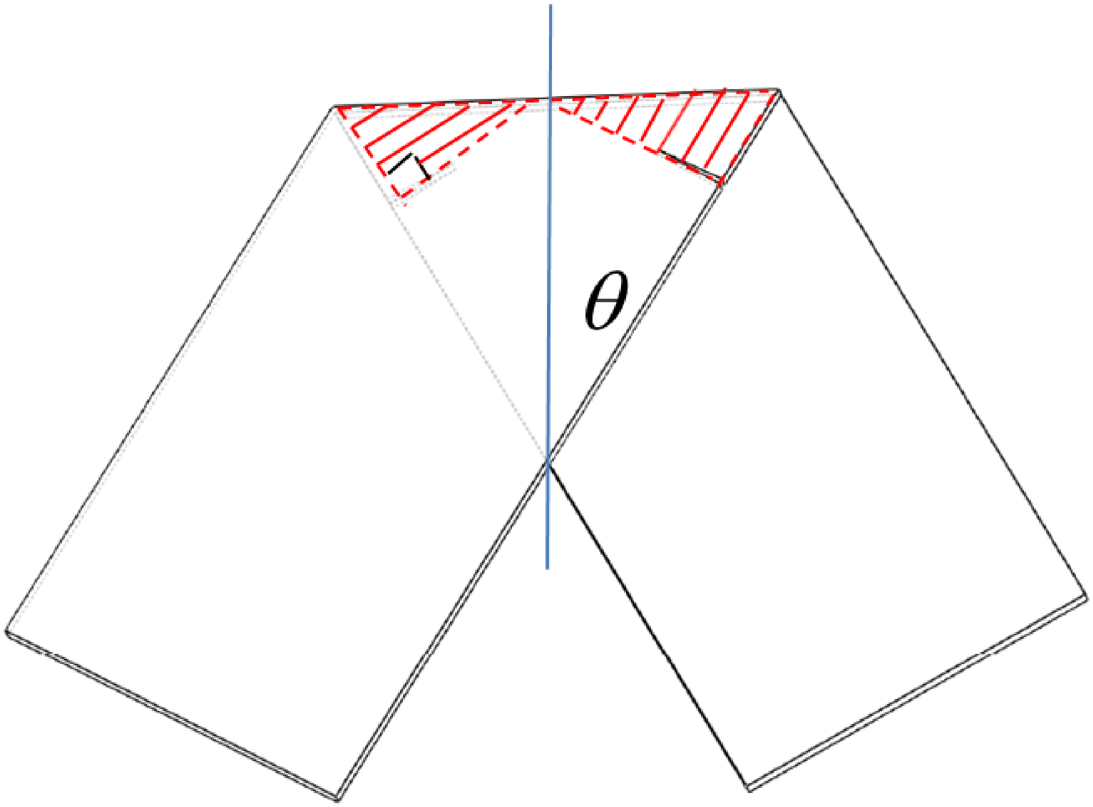

Supplementary Figure S7. Plastic zone generated during "plastic rolling".

For plastic rolling, β, the dissipated plastic energy density, was calculated by simulating folding a thin foil by a prescribed folding thickness. This problem was modeled by bending a thin film around a rigid circular die (Supplementary Fig. S8). The diameter of the rigid circular die corresponds to the folding thickness. The material parameters of Al were used in the analysis. Contact was defined between the deformable thin foil and the rigid die. 1,571 B22 (3-node quadratic beam) elements are used in the analysis. Once the thin foil enters the plastic zone, the plastic energy density can then be calculated.

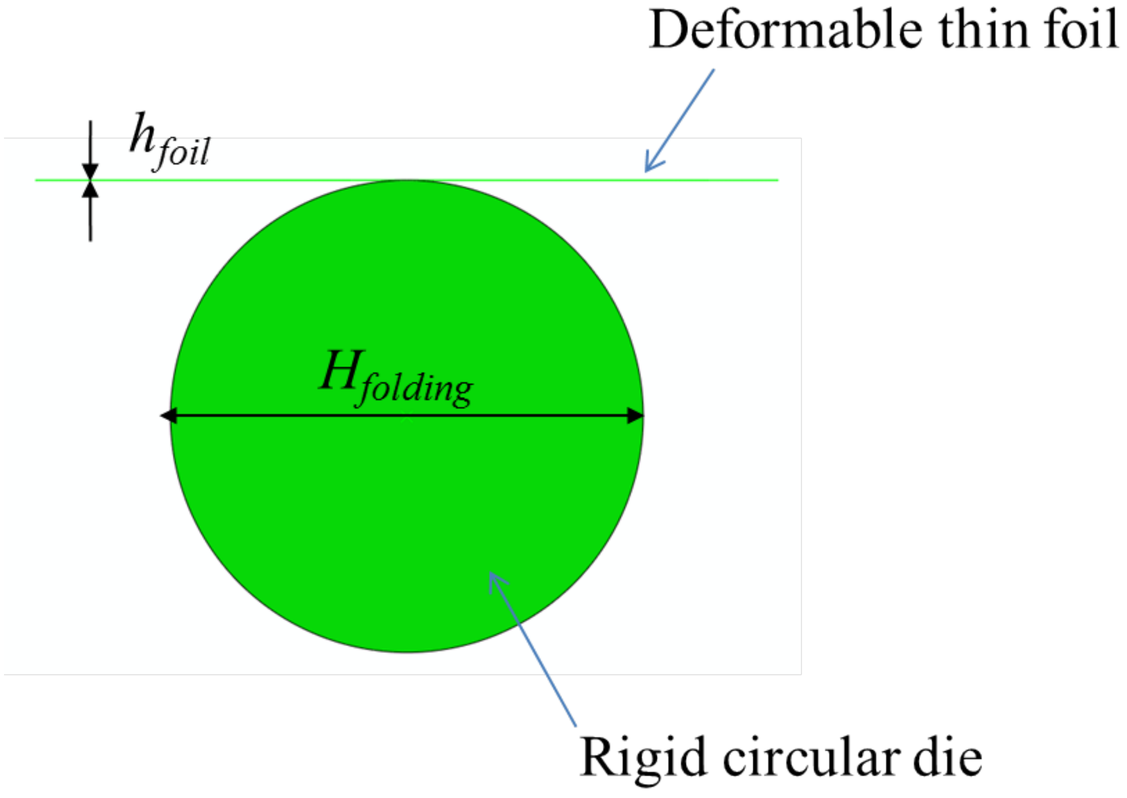

Supplementary Figure S8. Plastic zone generated during "plastic rolling".

Supplementary Fig. S9 shows β as a function of the ratio between the folding thickness and foil thickness $H_{folding}/h_{foil}$. It is found that as the ratio $H_{folding}/h_{foil}$ increases, β decreases. For the real battery setup, Al foil is 10 μm in thickness, while the entire battery cell is 500 μm - 800 μm in thickness depending on the mass loading of the active materials, which gives the ratio $H_{folding}/h_{foil}$ about 50 to 80. Within this range, Supplementary Fig. S9 shows that β is on the order of 1 MPa.

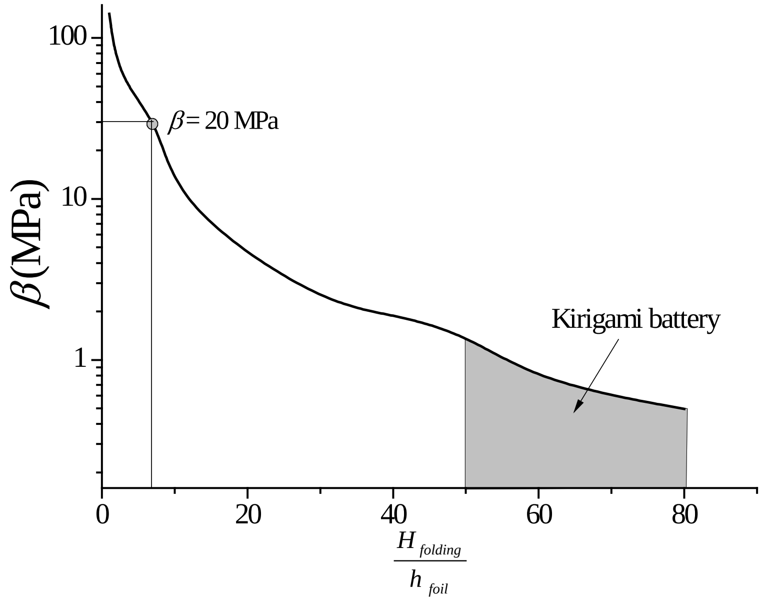

Supplementary Figure S9. The dissipated plastic energy per area (β) as a function of the extent of the folding crease that is characterized by the ratio between folding thickness and foil thickness.

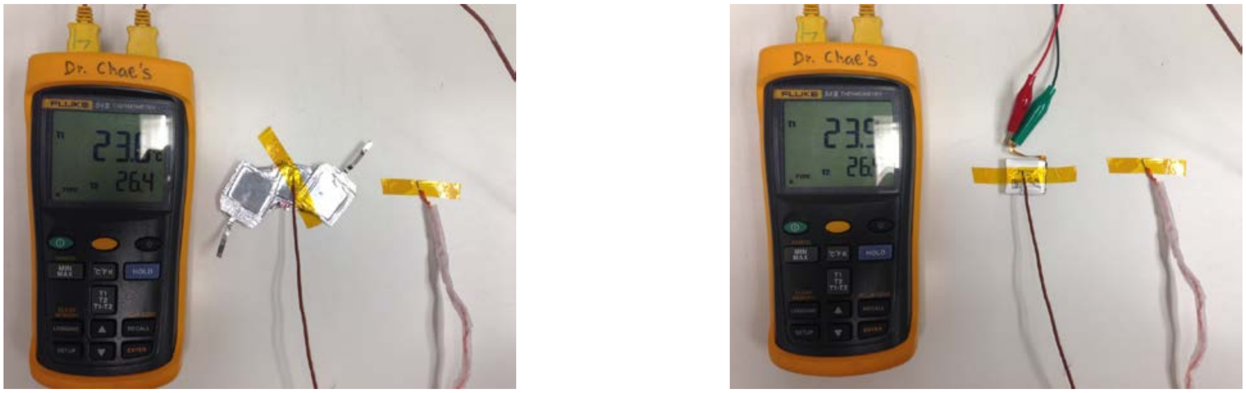

(a) (b)

Supplementary Figure S10. Thermal test of Kirigami battery and Samsung Gear 2 bulky battery. (a) Kirgami battery discharged at 48mA for one hour. **(b)** Samsung Gear 2 bulky battery discharged at 48mA for one hour.

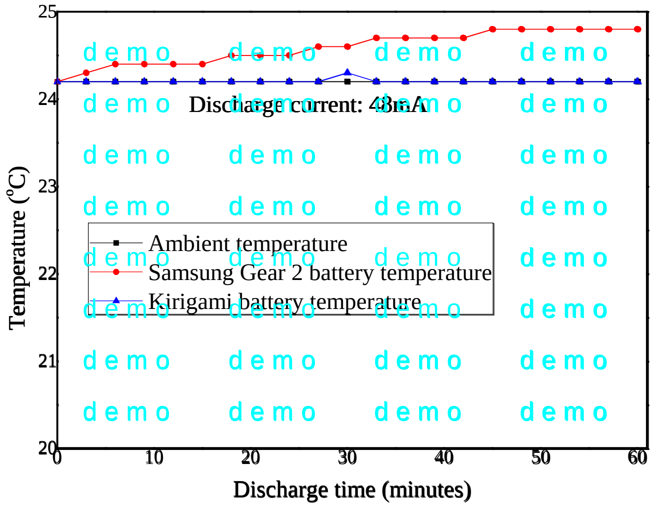

Supplementary Figure S11. Thermal test result of Kirigami battery and Samsung Gear 2 bulky battery.
